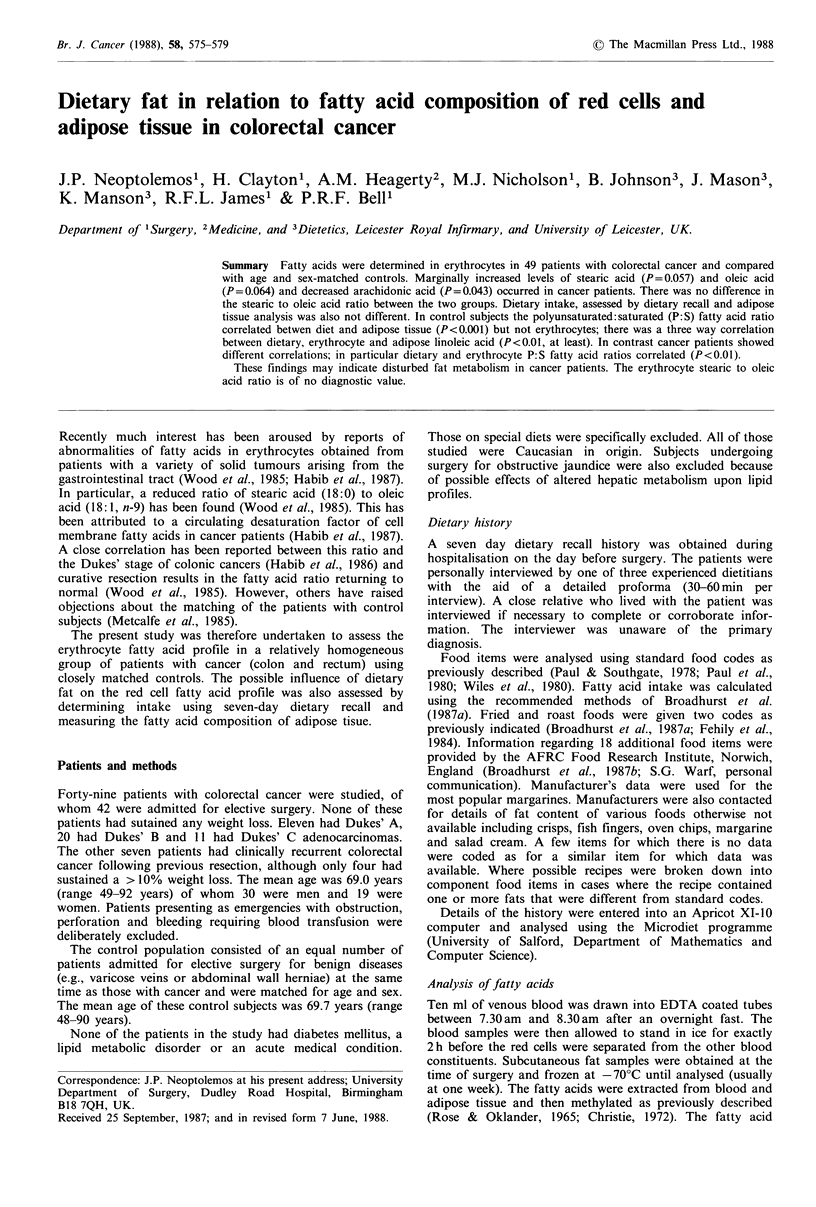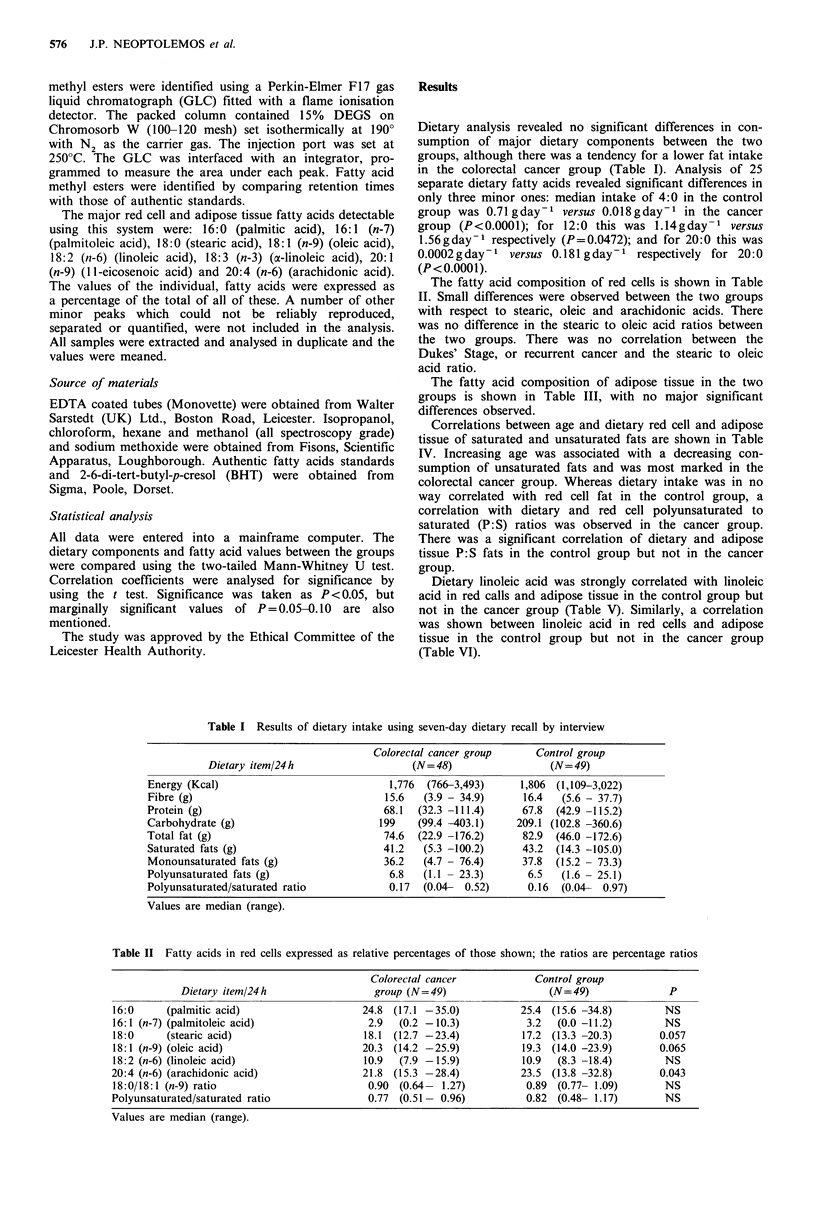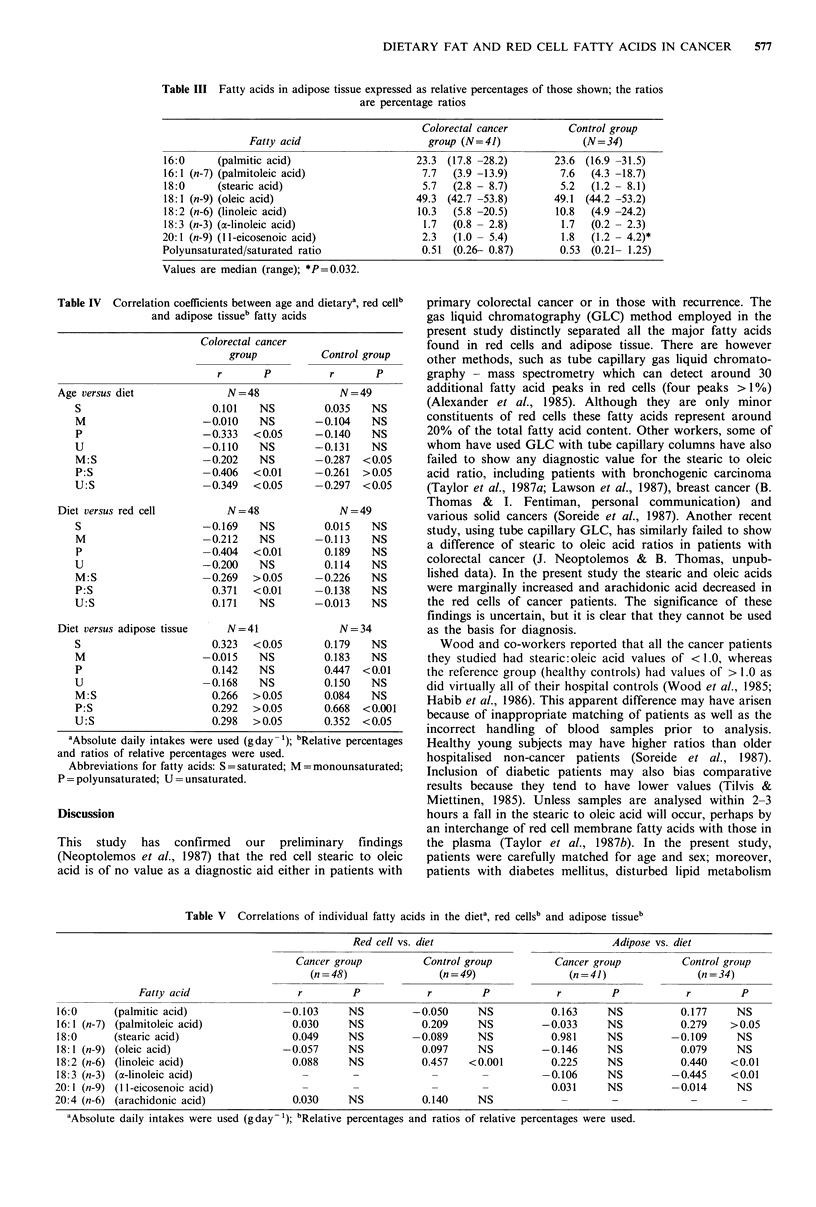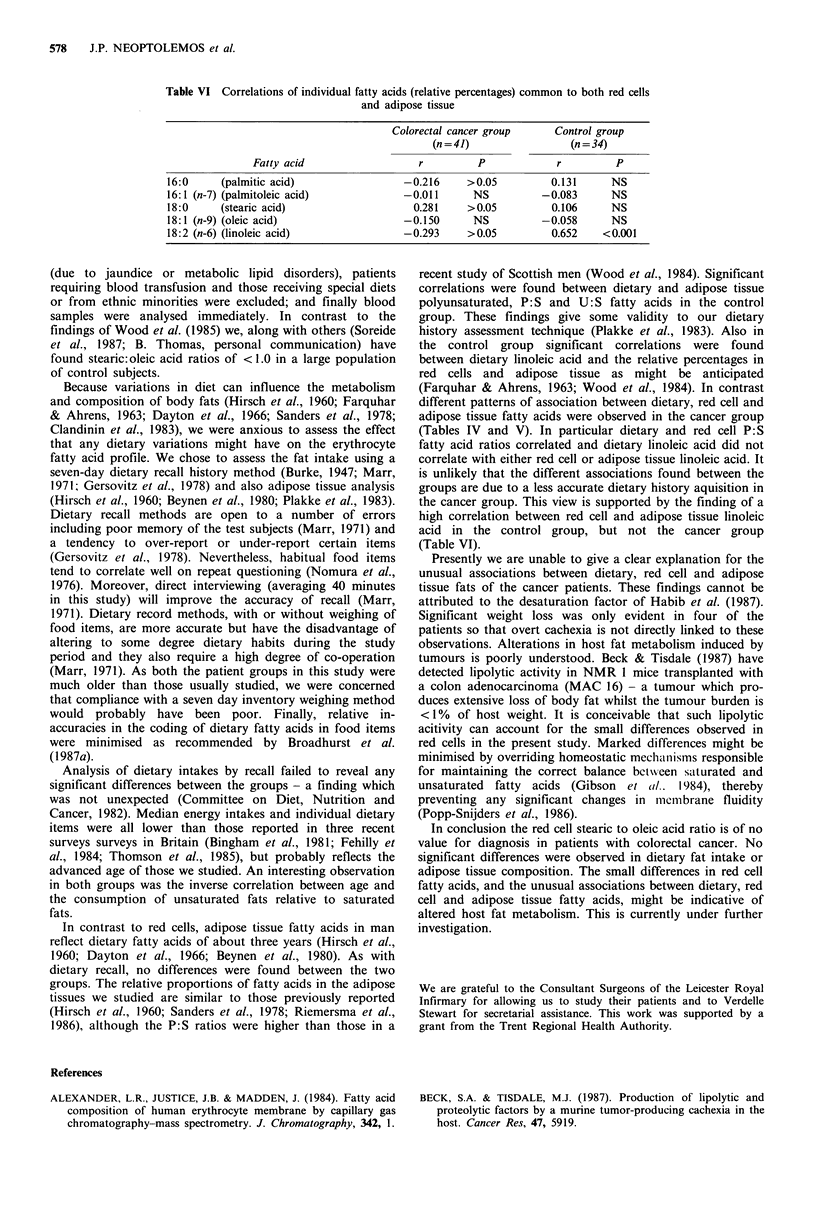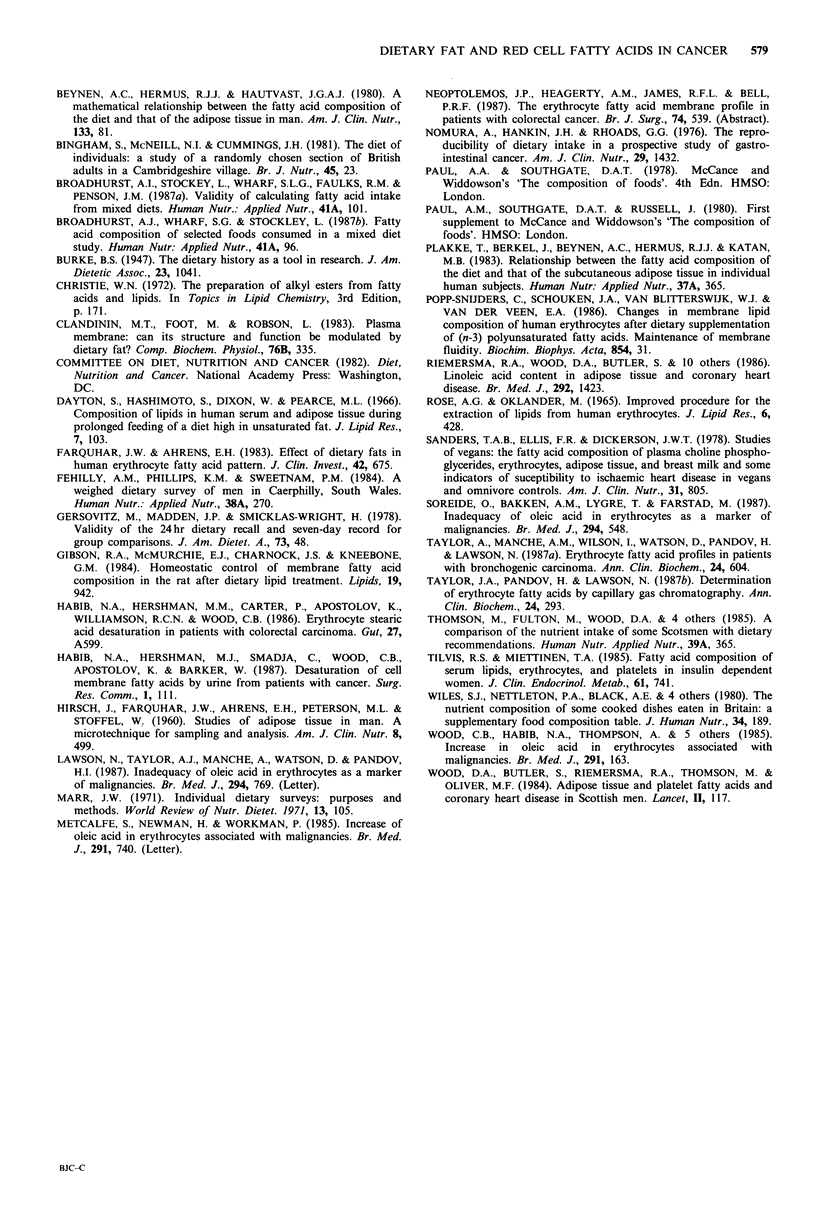# Dietary fat in relation to fatty acid composition of red cells and adipose tissue in colorectal cancer.

**DOI:** 10.1038/bjc.1988.262

**Published:** 1988-11

**Authors:** J. P. Neoptolemos, H. Clayton, A. M. Heagerty, M. J. Nicholson, B. Johnson, J. Mason, K. Manson, R. F. James, P. R. Bell

**Affiliations:** Department of Surgery, Leicester Royal Infirmary, UK.

## Abstract

Fatty acids were determined in erthrocytes in 49 patients with colorectal cancer and compared with age and sex-matched controls. Marginally increased levels of stearic acid (P = 0.057) and oleic acid (P = 0.064) and decreased arachidonic acid (P = 0.043) occurred in cancer patients. There was no difference in the stearic to oleic acid ratio between the two groups. Dietary intake, assessed by dietary recall and adipose tissue analysis was also not different. In control subjects the polyunsaturated:saturated (P:S) fatty acid ratio correlated between diet and adipose tissue (P less than 0.01, at least). In contrast cancer patients showed different correlations; in particular dietary and erythrocyte P:S fatty acid ratios correlated (P less than 0.01). These findings may indicate disturbed fat metabolism in cancer patients. The erythrocyte stearic to oleic acid ratio is of no diagnostic value.


*Br. J. Cancer* (1988), **58**, 575–579 © The Macmillan Press Ltd., 1988

### Dietary fat in relation to fatty acid composition of red cells and adipose tissue in colorectal cancer

J.P. Neoptolemos[1], H. Clayton[1], A.M. Heagerty[2], M.J. Nicholson[1], B. Johnson[3], J. Mason[3], K. Manson[3], R.F.L. James[1] & P.R.F. Bell[1]

*Department of [1]Surgery, [2]Medicine, and [3]Dietetics, Leicester Royal Infirmary, and University of Leicester, UK.*

**Summary** Fatty acids were determined in erythrocytes in 49 patients with colorectal cancer and compared with age and sex-matched controls. Marginally increased levels of stearic acid ($P=0.057$) and oleic acid ($P=0.064$) and decreased arachidonic acid ($P=0.043$) occurred in cancer patients. There was no difference in the stearic to oleic acid ratio between the two groups. Dietary intake, assessed by dietary recall and adipose tissue analysis was also not different. In control subjects the polyunsaturated:saturated (P:S) fatty acid ratio correlated betwen diet and adipose tissue ($P<0.001$) but not erythrocytes; there was a three way correlation between dietary, erythrocyte and adipose linoleic acid ($P<0.01$, at least). In contrast cancer patients showed different correlations; in particular dietary and erythrocyte P:S fatty acid ratios correlated ($P<0.01$).

These findings may indicate disturbed fat metabolism in cancer patients. The erythrocyte stearic to oleic acid ratio is of no diagnostic value.

Recently much interest has been aroused by reports of abnormalities of fatty acids in erythrocytes obtained from patients with a variety of solid tumours arising from the gastrointestinal tract (Wood *et al.*, 1985; Habib *et al.*, 1987). In particular, a reduced ratio of stearic acid (18:0) to oleic acid (18:1, *n*-9) has been found (Wood *et al.*, 1985). This has been attributed to a circulating desaturation factor of cell membrane fatty acids in cancer patients (Habib *et al.*, 1987). A close correlation has been reported between this ratio and the Dukes' stage of colonic cancers (Habib *et al.*, 1986) and curative resection results in the fatty acid ratio returning to normal (Wood *et al.*, 1985). However, others have raised objections about the matching of the patients with control subjects (Metcalfe *et al.*, 1985).

The present study was therefore undertaken to assess the erythrocyte fatty acid profile in a relatively homogeneous group of patients with cancer (colon and rectum) using closely matched controls. The possible influence of dietary fat on the red cell fatty acid profile was also assessed by determining intake using seven-day dietary recall and measuring the fatty acid composition of adipose tissue.

### Patients and methods

Forty-nine patients with colorectal cancer were studied, of whom 42 were admitted for elective surgery. None of these patients had sutained any weight loss. Eleven had Dukes' A, 20 had Dukes' B and 11 had Dukes' C adenocarcinomas. The other seven patients had clinically recurrent colorectal cancer following previous resection, although only four had sustained a >10% weight loss. The mean age was 69.0 years (range 49–92 years) of whom 30 were men and 19 were women. Patients presenting as emergencies with obstruction, perforation and bleeding requiring blood transfusion were deliberately excluded.

The control population consisted of an equal number of patients admitted for elective surgery for benign diseases (e.g., varicose veins or abdominal wall herniae) at the same time as those with cancer and were matched for age and sex. The mean age of these control subjects was 69.7 years (range 48–90 years).

None of the patients in the study had diabetes mellitus, a lipid metabolic disorder or an acute medical condition. Those on special diets were specifically excluded. All of those studied were Caucasian in origin. Subjects undergoing surgery for obstructive jaundice were also excluded because of possible effects of altered hepatic metabolism upon lipid profiles.

### *Dietary history*

A seven day dietary recall history was obtained during hospitalisation on the day before surgery. The patients were personally interviewed by one of three experienced dietitians with the aid of a detailed proforma (30–60 min per interview). A close relative who lived with the patient was interviewed if necessary to complete or corroborate information. The interviewer was unaware of the primary diagnosis.

Food items were analysed using standard food codes as previously described (Paul & Southgate, 1978; Paul *et al.*, 1980; Wiles *et al.*, 1980). Fatty acid intake was calculated using the recommended methods of Broadhurst *et al.* (1987*a*). Fried and roast foods were given two codes as previously indicated (Broadhurst *et al.*, 1987*a*; Fehily *et al.*, 1984). Information regarding 18 additional food items were provided by the AFRC Food Research Institute, Norwich, England (Broadhurst *et al.*, 1987*b*; S.G. Warf, personal communication). Manufacturer's data were used for the most popular margarines. Manufacturers were also contacted for details of fat content of various foods otherwise not available including crisps, fish fingers, oven chips, margarine and salad cream. A few items for which there is no data were coded as for a similar item for which data was available. Where possible recipes were broken down into component food items in cases where the recipe contained one or more fats that were different from standard codes.

Details of the history were entered into an Apricot XI-10 computer and analysed using the Microdiet programme (University of Salford, Department of Mathematics and Computer Science).

### *Analysis of fatty acids*

Ten ml of venous blood was drawn into EDTA coated tubes between 7.30 am and 8.30 am after an overnight fast. The blood samples were then allowed to stand in ice for exactly 2 h before the red cells were separated from the other blood constituents. Subcutaneous fat samples were obtained at the time of surgery and frozen at −70°C until analysed (usually at one week). The fatty acids were extracted from blood and adipose tissue and then methylated as previously described (Rose & Oklander, 1965; Christie, 1972). The fatty acid

Correspondence: J.P. Neoptolemos at his present address; University Department of Surgery, Dudley Road Hospital, Birmingham B18 7QH, UK.
Received 25 September, 1987; and in revised form 7 June, 1988.

methyl esters were identified using a Perkin-Elmer F17 gas liquid chromatograph (GLC) fitted with a flame ionisation detector. The packed column contained 15% DEGS on Chromosorb W (100–120 mesh) set isothermically at 190° with $N_2$ as the carrier gas. The injection port was set at 250°C. The GLC was interfaced with an integrator, programmed to measure the area under each peak. Fatty acid methyl esters were identified by comparing retention times with those of authentic standards.

The major red cell and adipose tissue fatty acids detectable using this system were: 16:0 (palmitic acid), 16:1 (*n*-7) (palmitoleic acid), 18:0 (stearic acid), 18:1 (*n*-9) (oleic acid), 18:2 (*n*-6) (linoleic acid), 18:3 (*n*-3) (α-linoleic acid), 20:1 (*n*-9) (11-eicosenoic acid) and 20:4 (*n*-6) (arachidonic acid). The values of the individual, fatty acids were expressed as a percentage of the total of all of these. A number of other minor peaks which could not be reliably reproduced, separated or quantified, were not included in the analysis. All samples were extracted and analysed in duplicate and the values were meaned.

*Source of materials*

EDTA coated tubes (Monovette) were obtained from Walter Sarstedt (UK) Ltd., Boston Road, Leicester. Isopropanol, chloroform, hexane and methanol (all spectroscopy grade) and sodium methoxide were obtained from Fisons, Scientific Apparatus, Loughborough. Authentic fatty acids standards and 2-6-di-tert-butyl-*p*-cresol (BHT) were obtained from Sigma, Poole, Dorset.

*Statistical analysis*

All data were entered into a mainframe computer. The dietary components and fatty acid values between the groups were compared using the two-tailed Mann-Whitney U test. Correlation coefficients were analysed for significance by using the *t* test. Significance was taken as $P<0.05$, but marginally significant values of $P=0.05$–$0.10$ are also mentioned.

The study was approved by the Ethical Committee of the Leicester Health Authority.

### Results

Dietary analysis revealed no significant differences in consumption of major dietary components between the two groups, although there was a tendency for a lower fat intake in the colorectal cancer group (Table I). Analysis of 25 separate dietary fatty acids revealed significant differences in only three minor ones: median intake of 4:0 in the control group was 0.71 g day$^{-1}$ *versus* 0.018 g day$^{-1}$ in the cancer group ($P<0.0001$); for 12:0 this was 1.14 g day$^{-1}$ *versus* 1.56 g day$^{-1}$ respectively ($P=0.0472$); and for 20:0 this was 0.0002 g day$^{-1}$ *versus* 0.181 g day$^{-1}$ respectively for 20:0 ($P<0.0001$).

The fatty acid composition of red cells is shown in Table II. Small differences were observed between the two groups with respect to stearic, oleic and arachidonic acids. There was no difference in the stearic to oleic acid ratios between the two groups. There was no correlation between the Dukes' Stage, or recurrent cancer and the stearic to oleic acid ratio.

The fatty acid composition of adipose tissue in the two groups is shown in Table III, with no major significant differences observed.

Correlations between age and dietary red cell and adipose tissue of saturated and unsaturated fats are shown in Table IV. Increasing age was associated with a decreasing consumption of unsaturated fats and was most marked in the colorectal cancer group. Whereas dietary intake was in no way correlated with red cell fat in the control group, a correlation with dietary and red cell polyunsaturated to saturated (P:S) ratios was observed in the cancer group. There was a significant correlation of dietary and adipose tissue P:S fats in the control group but not in the cancer group.

Dietary linoleic acid was strongly correlated with linoleic acid in red calls and adipose tissue in the control group but not in the cancer group (Table V). Similarly, a correlation was shown between linoleic acid in red cells and adipose tissue in the control group but not in the cancer group (Table VI).

**Table I** Results of dietary intake using seven-day dietary recall by interview

| *Dietary item/24 h* | *Colorectal cancer group (N=48)* | *Control group (N=49)* |
|---|---|---|
| Energy (Kcal) | 1,776 (766–3,493) | 1,806 (1,109–3,022) |
| Fibre (g) | 15.6 (3.9 - 34.9) | 16.4 (5.6 - 37.7) |
| Protein (g) | 68.1 (32.3 -111.4) | 67.8 (42.9 -115.2) |
| Carbohydrate (g) | 199 (99.4 -403.1) | 209.1 (102.8 -360.6) |
| Total fat (g) | 74.6 (22.9 -176.2) | 82.9 (46.0 -172.6) |
| Saturated fats (g) | 41.2 (5.3 -100.2) | 43.2 (14.3 -105.0) |
| Monounsaturated fats (g) | 36.2 (4.7 - 76.4) | 37.8 (15.2 - 73.3) |
| Polyunsaturated fats (g) | 6.8 (1.1 - 23.3) | 6.5 (1.6 - 25.1) |
| Polyunsaturated/saturated ratio | 0.17 (0.04– 0.52) | 0.16 (0.04– 0.97) |

Values are median (range).

**Table II** Fatty acids in red cells expressed as relative percentages of those shown; the ratios are percentage ratios

| *Dietary item/24 h* | *Colorectal cancer group (N=49)* | *Control group (N=49)* | *P* |
|---|---|---|---|
| 16:0 (palmitic acid) | 24.8 (17.1 −35.0) | 25.4 (15.6 -34.8) | NS |
| 16:1 (*n*-7) (palmitoleic acid) | 2.9 (0.2 −10.3) | 3.2 (0.0 -11.2) | NS |
| 18:0 (stearic acid) | 18.1 (12.7 −23.4) | 17.2 (13.3 -20.3) | 0.057 |
| 18:1 (*n*-9) (oleic acid) | 20.3 (14.2 −25.9) | 19.3 (14.0 -23.9) | 0.065 |
| 18:2 (*n*-6) (linoleic acid) | 10.9 (7.9 −15.9) | 10.9 (8.3 -18.4) | NS |
| 20:4 (*n*-6) (arachidonic acid) | 21.8 (15.3 −28.4) | 23.5 (13.8 -32.8) | 0.043 |
| 18:0/18:1 (*n*-9) ratio | 0.90 (0.64– 1.27) | 0.89 (0.77– 1.09) | NS |
| Polyunsaturated/saturated ratio | 0.77 (0.51– 0.96) | 0.82 (0.48– 1.17) | NS |

Values are median (range).

**Table III** Fatty acids in adipose tissue expressed as relative percentages of those shown; the ratios are percentage ratios

| Fatty acid | Colorectal cancer group (N=41) | Control group (N=34) |
|---|---|---|
| 16:0 (palmitic acid) | 23.3 (17.8 -28.2) | 23.6 (16.9 -31.5) |
| 16:1 (n-7) (palmitoleic acid) | 7.7 (3.9 -13.9) | 7.6 (4.3 -18.7) |
| 18:0 (stearic acid) | 5.7 (2.8 - 8.7) | 5.2 (1.2 - 8.1) |
| 18:1 (n-9) (oleic acid) | 49.3 (42.7 -53.8) | 49.1 (44.2 -53.2) |
| 18:2 (n-6) (linoleic acid) | 10.3 (5.8 -20.5) | 10.8 (4.9 -24.2) |
| 18:3 (n-3) (α-linoleic acid) | 1.7 (0.8 - 2.8) | 1.7 (0.2 - 2.3) |
| 20:1 (n-9) (11-eicosenoic acid) | 2.3 (1.0 - 5.4) | 1.8 (1.2 - 4.2)* |
| Polyunsaturated/saturated ratio | 0.51 (0.26- 0.87) | 0.53 (0.21- 1.25) |

Values are median (range); *$P=0.032$.

**Table IV** Correlation coefficients between age and dietary[a], red cell[b] and adipose tissue[b] fatty acids

| | Colorectal cancer group | | Control group | |
|---|---|---|---|---|
| | r | P | r | P |
| Age *versus* diet | N=48 | | N=49 | |
| S | 0.101 | NS | 0.035 | NS |
| M | −0.010 | NS | −0.104 | NS |
| P | −0.333 | <0.05 | −0.140 | NS |
| U | −0.110 | NS | −0.131 | NS |
| M:S | −0.202 | NS | −0.287 | <0.05 |
| P:S | −0.406 | <0.01 | −0.261 | >0.05 |
| U:S | −0.349 | <0.05 | −0.297 | <0.05 |
| Diet *versus* red cell | N=48 | | N=49 | |
| S | −0.169 | NS | 0.015 | NS |
| M | −0.212 | NS | −0.113 | NS |
| P | −0.404 | <0.01 | 0.189 | NS |
| U | −0.200 | NS | 0.114 | NS |
| M:S | −0.269 | >0.05 | −0.226 | NS |
| P:S | 0.371 | <0.01 | −0.138 | NS |
| U:S | 0.171 | NS | −0.013 | NS |
| Diet *versus* adipose tissue | N=41 | | N=34 | |
| S | 0.323 | <0.05 | 0.179 | NS |
| M | −0.015 | NS | 0.183 | NS |
| P | 0.142 | NS | 0.447 | <0.01 |
| U | −0.168 | NS | 0.150 | NS |
| M:S | 0.266 | >0.05 | 0.084 | NS |
| P:S | 0.292 | >0.05 | 0.668 | <0.001 |
| U:S | 0.298 | >0.05 | 0.352 | <0.05 |

[a]Absolute daily intakes were used ($g\,day^{-1}$); [b]Relative percentages and ratios of relative percentages were used.

Abbreviations for fatty acids: S = saturated; M = monounsaturated; P = polyunsaturated; U = unsaturated.

### Discussion

This study has confirmed our preliminary findings (Neoptolemos *et al.*, 1987) that the red cell stearic to oleic acid is of no value as a diagnostic aid either in patients with primary colorectal cancer or in those with recurrence. The gas liquid chromatography (GLC) method employed in the present study distinctly separated all the major fatty acids found in red cells and adipose tissue. There are however other methods, such as tube capillary gas liquid chromatography – mass spectrometry which can detect around 30 additional fatty acid peaks in red cells (four peaks >1%) (Alexander *et al.*, 1985). Although they are only minor constituents of red cells these fatty acids represent around 20% of the total fatty acid content. Other workers, some of whom have used GLC with tube capillary columns have also failed to show any diagnostic value for the stearic to oleic acid ratio, including patients with bronchogenic carcinoma (Taylor *et al.*, 1987*a*; Lawson *et al.*, 1987), breast cancer (B. Thomas & I. Fentiman, personal communication) and various solid cancers (Soreide *et al.*, 1987). Another recent study, using tube capillary GLC, has similarly failed to show a difference of stearic to oleic acid ratios in patients with colorectal cancer (J. Neoptolemos & B. Thomas, unpublished data). In the present study the stearic and oleic acids were marginally increased and arachidonic acid decreased in the red cells of cancer patients. The significance of these findings is uncertain, but it is clear that they cannot be used as the basis for diagnosis.

Wood and co-workers reported that all the cancer patients they studied had stearic:oleic acid values of <1.0, whereas the reference group (healthy controls) had values of >1.0 as did virtually all of their hospital controls (Wood *et al.*, 1985; Habib *et al.*, 1986). This apparent difference may have arisen because of inappropriate matching of patients as well as the incorrect handling of blood samples prior to analysis. Healthy young subjects may have higher ratios than older hospitalised non-cancer patients (Soreide *et al.*, 1987). Inclusion of diabetic patients may also bias comparative results because they tend to have lower values (Tilvis & Miettinen, 1985). Unless samples are analysed within 2–3 hours a fall in the stearic to oleic acid will occur, perhaps by an interchange of red cell membrane fatty acids with those in the plasma (Taylor *et al.*, 1987*b*). In the present study, patients were carefully matched for age and sex; moreover, patients with diabetes mellitus, disturbed lipid metabolism

**Table V** Correlations of individual fatty acids in the diet[a], red cells[b] and adipose tissue[b]

| Fatty acid | Red cell vs. diet: Cancer group (n=48) r | P | Red cell vs. diet: Control group (n=49) r | P | Adipose vs. diet: Cancer group (n=41) r | P | Adipose vs. diet: Control group (n=34) r | P |
|---|---|---|---|---|---|---|---|---|
| 16:0 (palmitic acid) | −0.103 | NS | −0.050 | NS | 0.163 | NS | 0.177 | NS |
| 16:1 (n-7) (palmitoleic acid) | 0.030 | NS | 0.209 | NS | −0.033 | NS | 0.279 | >0.05 |
| 18:0 (stearic acid) | 0.049 | NS | −0.089 | NS | 0.981 | NS | −0.109 | NS |
| 18:1 (n-9) (oleic acid) | −0.057 | NS | 0.097 | NS | −0.146 | NS | 0.079 | NS |
| 18:2 (n-6) (linoleic acid) | 0.088 | NS | 0.457 | <0.001 | 0.225 | NS | 0.440 | <0.01 |
| 18:3 (n-3) (α-linoleic acid) | – | – | – | – | −0.106 | NS | −0.445 | <0.01 |
| 20:1 (n-9) (11-eicosenoic acid) | – | – | – | – | 0.031 | NS | −0.014 | NS |
| 20:4 (n-6) (arachidonic acid) | 0.030 | NS | 0.140 | NS | – | – | – | – |

[a]Absolute daily intakes were used ($g\,day^{-1}$); [b]Relative percentages and ratios of relative percentages were used.

**Table VI** Correlations of individual fatty acids (relative percentages) common to both red cells and adipose tissue

| Fatty acid | *Colorectal cancer group (n=41)* | | *Control group (n=34)* | |
|---|---|---|---|---|
| | *r* | *P* | *r* | *P* |
| 16:0 (palmitic acid) | −0.216 | >0.05 | 0.131 | NS |
| 16:1 (*n*-7) (palmitoleic acid) | −0.011 | NS | −0.083 | NS |
| 18:0 (stearic acid) | 0.281 | >0.05 | 0.106 | NS |
| 18:1 (*n*-9) (oleic acid) | −0.150 | NS | −0.058 | NS |
| 18:2 (*n*-6) (linoleic acid) | −0.293 | >0.05 | 0.652 | <0.001 |

(due to jaundice or metabolic lipid disorders), patients requiring blood transfusion and those receiving special diets or from ethnic minorities were excluded; and finally blood samples were analysed immediately. In contrast to the findings of Wood *et al.* (1985) we, along with others (Soreide *et al.*, 1987; B. Thomas, personal communication) have found stearic:oleic acid ratios of <1.0 in a large population of control subjects.

Because variations in diet can influence the metabolism and composition of body fats (Hirsch *et al.*, 1960; Farquhar & Ahrens, 1963; Dayton *et al.*, 1966; Sanders *et al.*, 1978; Clandinin *et al.*, 1983), we were anxious to assess the effect that any dietary variations might have on the erythrocyte fatty acid profile. We chose to assess the fat intake using a seven-day dietary recall history method (Burke, 1947; Marr, 1971; Gersovitz *et al.*, 1978) and also adipose tissue analysis (Hirsch *et al.*, 1960; Beynen *et al.*, 1980; Plakke *et al.*, 1983). Dietary recall methods are open to a number of errors including poor memory of the test subjects (Marr, 1971) and a tendency to over-report or under-report certain items (Gersovitz *et al.*, 1978). Nevertheless, habitual food items tend to correlate well on repeat questioning (Nomura *et al.*, 1976). Moreover, direct interviewing (averaging 40 minutes in this study) will improve the accuracy of recall (Marr, 1971). Dietary record methods, with or without weighing of food items, are more accurate but have the disadvantage of altering to some degree dietary habits during the study period and they also require a high degree of co-operation (Marr, 1971). As both the patient groups in this study were much older than those usually studied, we were concerned that compliance with a seven day inventory weighing method would probably have been poor. Finally, relative inaccuracies in the coding of dietary fatty acids in food items were minimised as recommended by Broadhurst *et al.* (1987*a*).

Analysis of dietary intakes by recall failed to reveal any significant differences between the groups – a finding which was not unexpected (Committee on Diet, Nutrition and Cancer, 1982). Median energy intakes and individual dietary items were all lower than those reported in three recent surveys surveys in Britain (Bingham *et al.*, 1981; Fehilly *et al.*, 1984; Thomson *et al.*, 1985), but probably reflects the advanced age of those we studied. An interesting observation in both groups was the inverse correlation between age and the consumption of unsaturated fats relative to saturated fats.

In contrast to red cells, adipose tissue fatty acids in man reflect dietary fatty acids of about three years (Hirsch *et al.*, 1960; Dayton *et al.*, 1966; Beynen *et al.*, 1980). As with dietary recall, no differences were found between the two groups. The relative proportions of fatty acids in the adipose tissues we studied are similar to those previously reported (Hirsch *et al.*, 1960; Sanders *et al.*, 1978; Riemersma *et al.*, 1986), although the P:S ratios were higher than those in a recent study of Scottish men (Wood *et al.*, 1984). Significant correlations were found between dietary and adipose tissue polyunsaturated, P:S and U:S fatty acids in the control group. These findings give some validity to our dietary history assessment technique (Plakke *et al.*, 1983). Also in the control group significant correlations were found between dietary linoleic acid and the relative percentages in red cells and adipose tissue as might be anticipated (Farquhar & Ahrens, 1963; Wood *et al.*, 1984). In contrast different patterns of association between dietary, red cell and adipose tissue fatty acids were observed in the cancer group (Tables IV and V). In particular dietary and red cell P:S fatty acid ratios correlated and dietary linoleic acid did not correlate with either red cell or adipose tissue linoleic acid. It is unlikely that the different associations found between the groups are due to a less accurate dietary history aquisition in the cancer group. This view is supported by the finding of a high correlation between red cell and adipose tissue linoleic acid in the control group, but not the cancer group (Table VI).

Presently we are unable to give a clear explanation for the unusual associations between dietary, red cell and adipose tissue fats of the cancer patients. These findings cannot be attributed to the desaturation factor of Habib *et al.* (1987). Significant weight loss was only evident in four of the patients so that overt cachexia is not directly linked to these observations. Alterations in host fat metabolism induced by tumours is poorly understood. Beck & Tisdale (1987) have detected lipolytic activity in NMR 1 mice transplanted with a colon adenocarcinoma (MAC 16) – a tumour which produces extensive loss of body fat whilst the tumour burden is <1% of host weight. It is conceivable that such lipolytic acitivity can account for the small differences observed in red cells in the present study. Marked differences might be minimised by overriding homeostatic mechanisms responsible for maintaining the correct balance between saturated and unsaturated fatty acids (Gibson *et al.*, 1984), thereby preventing any significant changes in membrane fluidity (Popp-Snijders *et al.*, 1986).

In conclusion the red cell stearic to oleic acid ratio is of no value for diagnosis in patients with colorectal cancer. No significant differences were observed in dietary fat intake or adipose tissue composition. The small differences in red cell fatty acids, and the unusual associations between dietary, red cell and adipose tissue fatty acids, might be indicative of altered host fat metabolism. This is currently under further investigation.

We are grateful to the Consultant Surgeons of the Leicester Royal Infirmary for allowing us to study their patients and to Verdelle Stewart for secretarial assistance. This work was supported by a grant from the Trent Regional Health Authority.

### References

ALEXANDER, L.R., JUSTICE, J.B. & MADDEN, J. (1984). Fatty acid composition of human erythrocyte membrane by capillary gas chromatography–mass spectrometry. *J. Chromatography*, **342**, 1.

BECK, S.A. & TISDALE, M.J. (1987). Production of lipolytic and proteolytic factors by a murine tumor-producing cachexia in the host. *Cancer Res*, **47**, 5919.

BEYNEN, A.C., HERMUS, R.J.J. & HAUTVAST, J.G.A.J. (1980). A mathematical relationship between the fatty acid composition of the diet and that of the adipose tissue in man. *Am. J. Clin. Nutr.*, **133,** 81.

BINGHAM, S., McNEILL, N.I. & CUMMINGS, J.H. (1981). The diet of individuals: a study of a randomly chosen section of British adults in a Cambridgeshire village. *Br. J. Nutr.*, **45,** 23.

BROADHURST, A.I., STOCKEY, L., WHARF, S.L.G., FAULKS, R.M. & PENSON, J.M. (1987*a*). Validity of calculating fatty acid intake from mixed diets. *Human Nutr.: Applied Nutr.*, **41A,** 101.

BROADHURST, A.J., WHARF, S.G. & STOCKLEY, L. (1987*b*). Fatty acid composition of selected foods consumed in a mixed diet study. *Human Nutr: Applied Nutr.*, **41A,** 96.

BURKE, B.S. (1947). The dietary history as a tool in research. *J. Am. Dietetic Assoc.*, **23,** 1041.

CHRISTIE, W.N. (1972). The preparation of alkyl esters from fatty acids and lipids. In *Topics in Lipid Chemistry*, 3rd Edition, p. 171.

CLANDININ, M.T., FOOT, M. & ROBSON, L. (1983). Plasma membrane: can its structure and function be modulated by dietary fat? *Comp. Biochem. Physiol.*, **76B,** 335.

COMMITTEE ON DIET, NUTRITION AND CANCER (1982). *Diet, Nutrition and Cancer*. National Academy Press: Washington, DC.

DAYTON, S., HASHIMOTO, S., DIXON, W. & PEARCE, M.L. (1966). Composition of lipids in human serum and adipose tissue during prolonged feeding of a diet high in unsaturated fat. *J. Lipid Res.*, **7,** 103.

FARQUHAR, J.W. & AHRENS, E.H. (1983). Effect of dietary fats in human erythrocyte fatty acid pattern. *J. Clin. Invest.*, **42,** 675.

FEHILLY, A.M., PHILLIPS, K.M. & SWEETNAM, P.M. (1984). A weighed dietary survey of men in Caerphilly, South Wales. *Human Nutr.: Applied Nutr.*, **38A,** 270.

GERSOVITZ, M., MADDEN, J.P. & SMICKLAS-WRIGHT, H. (1978). Validity of the 24 hr dietary recall and seven-day record for group comparisons. *J. Am. Dietet. A.*, **73,** 48.

GIBSON, R.A., McMURCHIE, E.J., CHARNOCK, J.S. & KNEEBONE, G.M. (1984). Homeostatic control of membrane fatty acid composition in the rat after dietary lipid treatment. *Lipids*, **19,** 942.

HABIB, N.A., HERSHMAN, M.M., CARTER, P., APOSTOLOV, K., WILLIAMSON, R.C.N. & WOOD, C.B. (1986). Erythrocyte stearic acid desaturation in patients with colorectal carcinoma. *Gut*, **27,** A599.

HABIB, N.A., HERSHMAN, M.J., SMADJA, C., WOOD, C.B., APOSTOLOV, K. & BARKER, W. (1987). Desaturation of cell membrane fatty acids by urine from patients with cancer. *Surg. Res. Comm.*, **1,** 111.

HIRSCH, J., FARQUHAR, J.W., AHRENS, E.H., PETERSON, M.L. & STOFFEL, W. (1960). Studies of adipose tissue in man. A microtechnique for sampling and analysis. *Am. J. Clin. Nutr.* **8,** 499.

LAWSON, N., TAYLOR, A.J., MANCHE, A., WATSON, D. & PANDOV, H.I. (1987). Inadequacy of oleic acid in erythrocytes as a marker of malignancies. *Br. Med. J.*, **294,** 769. (Letter).

MARR, J.W. (1971). Individual dietary surveys: purposes and methods. *World Review of Nutr. Dietet. 1971*, **13,** 105.

METCALFE, S., NEWMAN, H. & WORKMAN, P. (1985). Increase of oleic acid in erythrocytes associated with malignancies. *Br. Med. J.*, **291,** 740. (Letter).

NEOPTOLEMOS, J.P., HEAGERTY, A.M., JAMES, R.F.L. & BELL, P.R.F. (1987). The erythrocyte fatty acid membrane profile in patients with colorectal cancer. *Br. J. Surg.*, **74,** 539. (Abstract).

NOMURA, A., HANKIN, J.H. & RHOADS, G.G. (1976). The reproducibility of dietary intake in a prospective study of gastrointestinal cancer. *Am. J. Clin. Nutr.*, **29,** 1432.

PAUL, A.A. & SOUTHGATE, D.A.T. (1978). McCance and Widdowson's 'The composition of foods'. 4th Edn. HMSO: London.

PAUL, A.M., SOUTHGATE, D.A.T. & RUSSELL, J. (1980). First supplement to McCance and Widdowson's 'The composition of foods'. HMSO: London.

PLAKKE, T., BERKEL, J., BEYNEN, A.C., HERMUS, R.J.J. & KATAN, M.B. (1983). Relationship between the fatty acid composition of the diet and that of the subcutaneous adipose tissue in individual human subjects. *Human Nutr: Applied Nutr.*, **37A,** 365.

POPP-SNIJDERS, C., SCHOUKEN, J.A., VAN BLITTERSWIJK, W.J. & VAN DER VEEN, E.A. (1986). Changes in membrane lipid composition of human erythrocytes after dietary supplementation of (*n*-3) polyunsaturated fatty acids. Maintenance of membrane fluidity. *Biochim. Biophys. Acta*, **854,** 31.

RIEMERSMA, R.A., WOOD, D.A., BUTLER, S. & 10 others (1986). Linoleic acid content in adipose tissue and coronary heart disease. *Br. Med. J.*, **292,** 1423.

ROSE, A.G. & OKLANDER, M. (1965). Improved procedure for the extraction of lipids from human erythrocytes. *J. Lipid Res.*, **6,** 428.

SANDERS, T.A.B., ELLIS, F.R. & DICKERSON, J.W.T. (1978). Studies of vegans: the fatty acid composition of plasma choline phosphoglycerides, erythrocytes, adipose tissue, and breast milk and some indicators of suceptibility to ischaemic heart disease in vegans and omnivore controls. *Am. J. Clin. Nutr.*, **31,** 805.

SOREIDE, O., BAKKEN, A.M., LYGRE, T. & FARSTAD, M. (1987). Inadequacy of oleic acid in erythrocytes as a marker of malignancies. *Br. Med. J.*, **294,** 548.

TAYLOR, A., MANCHE, A.M., WILSON, I., WATSON, D., PANDOV, H. & LAWSON, N. (1987*a*). Erythrocyte fatty acid profiles in patients with bronchogenic carcinoma. *Ann. Clin. Biochem.*, **24,** 604.

TAYLOR, J.A., PANDOV, H. & LAWSON, N. (1987*b*). Determination of erythrocyte fatty acids by capillary gas chromatography. *Ann. Clin. Biochem.*, **24,** 293.

THOMSON, M., FULTON, M., WOOD, D.A. & 4 others (1985). A comparison of the nutrient intake of some Scotsmen with dietary recommendations. *Human Nutr. Applied Nutr.*, **39A,** 365.

TILVIS, R.S. & MIETTINEN, T.A. (1985). Fatty acid composition of serum lipids, erythrocytes, and platelets in insulin dependent women. *J. Clin. Endocrinol. Metab.*, **61,** 741.

WILES, S.J., NETTLETON, P.A., BLACK, A.E. & 4 others (1980). The nutrient composition of some cooked dishes eaten in Britain: a supplementary food composition table. *J. Human Nutr.*, **34,** 189.

WOOD, C.B., HABIB, N.A., THOMPSON, A. & 5 others (1985). Increase in oleic acid in erythrocytes associated with malignancies. *Br. Med. J.*, **291,** 163.

WOOD, D.A., BUTLER, S., RIEMERSMA, R.A., THOMSON, M. & OLIVER, M.F. (1984). Adipose tissue and platelet fatty acids and coronary heart disease in Scottish men. *Lancet*, **II,** 117.